# Supplementary figures and images for: Effect of sugar metabolite methylglyoxal on equine lamellar explants: An ex vivo model of laminitis
Source: PLoS One. 2021 Jul 27;16(7):e0253840. doi: 10.1371/journal.pone.0253840 (PMC8315528; doi:10.1371/journal.pone.0253840)

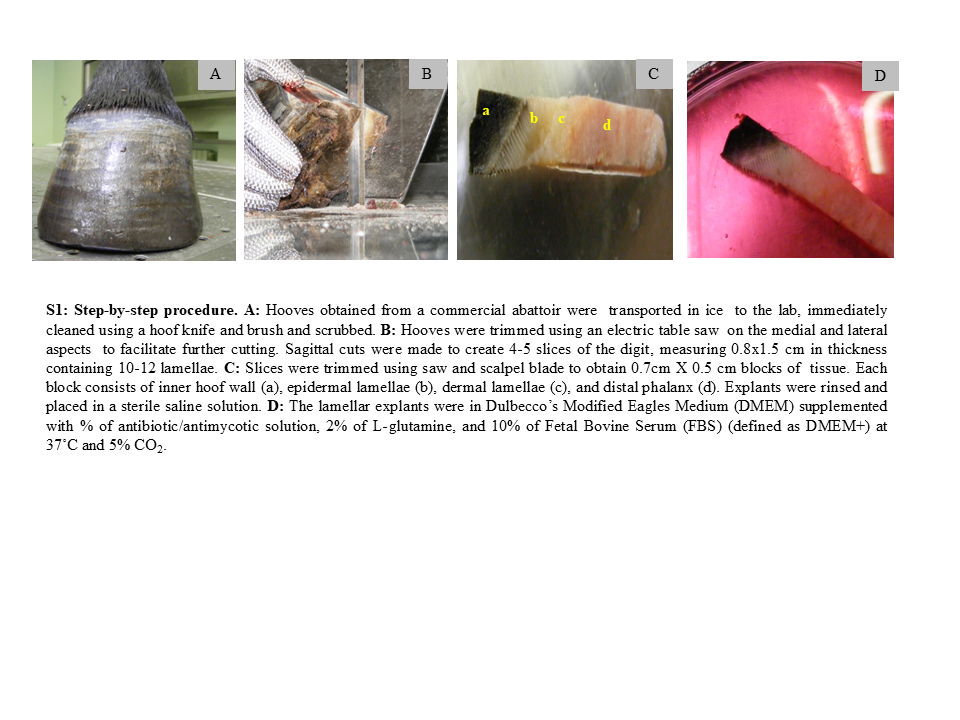

Supplement: S1 Fig — A: Hooves obtained from a commercial abattoir were transported in ice to the lab, immediately cleaned using a hoof knife and brush and scrubbed. B: Hooves were trimmed using an electric table saw on the medial and lateral aspects to facilitate further cutting. Sagittal cuts were made to create 4–5 slices of the digit, measuring 0.8x1.5 cm in thickness containing 10–12 lamellae. C: Slices were trimmed using saw and scalpel blade to obtain 0.7cm X 0.5 cm blocks of tissue. Each block consists of inner hoof wall (a), epidermal lamellae (b), dermal lamellae (c), and distal phalanx (d). Explants were rinsed and placed in a sterile saline solution. D: The lamellar explants were in Dulbecco’s Modified Eagles Medium (DMEM) supplemented with % of antibiotic/antimycotic solution, 2% of L-glutamine, and 10% of Fetal Bovine Serum (FBS) (defined as DMEM+) at 37°C and 5% CO2. (TIF) [file pone.0253840.s002.tif]
