# Supplementary material for: Effect of sugar metabolite methylglyoxal on equine lamellar explants: An ex vivo model of laminitis
Source: PLoS One. 2021 Jul 27;16(7):e0253840. doi: 10.1371/journal.pone.0253840 (PMC8315528; doi:10.1371/journal.pone.0253840)
Supplement: Attachment — Submitted filename: Response to Reviewers.docx [file pone.0253840.s003.docx]

**Response letter manuscript PONE-D-20-06143**

**Journal requirements:**

- The manuscript has been formatted according to Journal’s style
- The specification about funding has been added and the previous funding statement has been corrected
- The overlapping texts have been corrected according to the suggestions.
  - <https://www.ncbi.nlm.nih.gov/pmc/articles/PMC3605361/> 🡪 this reference (Miretti et al., 2017) has been added along the text and in reference list. Thank You for the suggestion because we forgot to cite a paper of one of the co-authors.
  - <https://onlinelibrary.wiley.com/doi/full/10.1002/dmrr.2811> 🡪 this paper was already present in the reference list and we added a new reference along the text.
- <https://beva.onlinelibrary.wiley.com/doi/abs/10.2746/042516406778400565> 🡪 we are really sorry but we can not add this reference because we did not consult it to write the paper. We don’t know why there’s an overlapping.
- <https://www.sciencedirect.com/science/article/abs/pii/S0165242711000201?via%3Dihub> 🡪 this paper was already present in the reference list and we added a new reference along the text.

**Reviewer 1**

Dear Reviewer, on behalf of all Authors, I would like to thank You for the time that you have spent to revise our manuscript. We appreciate Your efforts to improve our manuscript. We accepted all Your suggestions, and we provided the best answers that we could to the questions and comments that You addressed.

Here I provide a point-to-point response. When rephrasing was suggested, I have just stated “fixed”. When a longer answer was needed, I provided an explanation.

Line 1: fixed

**Abstract**

- Line 15: fixed
- Line 16: fixed
- Line 18: fixed
- Line 19: fixed
- Line 20: fixed
- Line 21: fixed
- Line 22: fixed
- Line 23: fixed
- Line 26: fixed
- Line 27: fixed

**Introduction**

- Line 33: fixed
- Line 37: fixed
- Line 38-39: fixed
- Line 41: fixed
- Line 46: fixed
- Line 48: fixed
- Line 49: fixed
- Line 50: fixed
- Line 52: fixed
- Line 54: fixed
- Line 56: fixed
- Line 58: fixed
- Line 59: fixed
- Line 60: fixed
- Line 61: fixed. To answer to the Reviewer’s question “ *has this substance been shown to appear in equine serum/plasma following induction of a carbohydrate overload model of laminitis? That would seem to be central to the premise here, given the proposed route of exposure of the lamellae to this substance in vivo*” some sentences were added in order to clarify (L61-69 in the revised version).
- Line 64-65: To answer to the Reviewer’s question “*Would include some context-specific criteria where lactate may serve as a biomarker of laminitis risk (not laminitis itself) - certain situations are associated with hyperlactatemia but not laminitis risk (such as intense aerobic exercise), so would restrict this in some way to situations in which this is more likely to be the case (e.g., sepsis)*” some sentences were added (L75-80 in the revised version).
- Line 67: fixed
- Line 70: fixed
- Line 72: fixed
- Line 77: fixed

**Material and methods**

- Line 81: According to Reviewer’s comment: “*the authors might comment in the discussion about how widely these data might be extrapolated to other breeds/types of horses*”, Authors provided a short explanation in discussion session (L 389-391 in the revised version).
- Line 82: fixed
- Line 84: fixed
- Line 88: To answer to the Reviewer’s questions: “*hind limb or front limb? List signs of laminitis that were excluded?*” Authors added that front limbs were collected (L 99 in the revised version) and wrote the evaluated signs and symptoms (L 103-104 in the revised version).
- Line 89: fixed
- Line 101: fixed in this line and along the text
- Line 114: fixed
- Lines 116-117: According to Reviewer’s comment: “would include how survival was evaluated here (not just in the Results section)” a short sentence was added (L 131-132 in the revised version).
- Line 117: fixed
- Line 130: fixed
- Lines 130-131: According to Reviewer’s comment: “I'm not sure what the authors are trying to say here, it's a bit unclear; consider rewording this sentence”, Authors rephrased (L 147-149 in the revised version).
- Line 136: fixed
- Line142: fixed
- Line 144: fixed
- Lines 146-147: According to Reviewer’s comment: ”*how long after the force testing did RNA extraction occur? Was RNA extracted from any samples that were not subjected to force testing? Can the authors comment on the likely influence of this testing on the mRNA concentrations of some of their target genes in the sample tissue (i.e., are they known to be influenced by stretch, if sufficient time had elapsed between force testing and RNA extraction)?*” Authors added some short sentences in lines 147-150 and 169-171 in order to explain that samples were immediately treated after the separation force test. According with reviewer's comment could be interesting evaluate changing in gene expression after the force test, but when the samples are maintained in culture condition for additional time after the test as reported by other studies (REF: Wu JH et al, Bone Joint Res. 2017 Mar;6(3):179-185; Qin TW, Sun YL, Thoreson AR, et al.. Effect of mechanical stimulation on bone marrow stromal cell-seeded tendon slice constructs: a potential engineered tendon patch for rotator cuff repair. Biomaterials 2015;51:43-50.The revitalisation of flexor tendon allografts with bone marrow stromal cells and mechanical stimulation: An ex vivo model revitalising flexor tendon allografts). In our case, we consider negligible the influence of the force test on the gene expression due to the closed timing between the test and RNA extraction.
- Line 157: fixed
- Line162: fixed
- Line 164: fixed
- Line 169: fixed
- Line 174: fixed
- Line 175: According to Reviewer’s question “*how was this determined*?” Authors answer that GAPDH was selected as housekeeping gene for the stable Cq values among the different experimental conditions. The correction is provided in lines 194-197 of the revised version.
- Line 177: fixed
- Line 179: fixed
- Line 187: Authors tried to provide the suggested correction. We hope to have interpreted well the comments.
- Line 190-193: fixed
- Line 192: According to Reviewer’s question: “How were the distributions of the histology score data analyzed?” the test’s name (L210-211 in the revised version) and a short explanation (L 269-270 in the revised version) were added.
- Line 196: fixed

**Results**

- Lines 200-201: According to Reviewer’s comment: *“Were any more sensitive/quantifiable measures of autolysis used to evaluate this tissue? This seems like a very subjective assessment.”*, Authors tried to rephrase and explain better (L 221 in the revised version).
- Line 202: According to Reviewer’s question “*Can the authors include a figure displaying representative histologic sections?”*, Authors provided pictures to show the differences between well preserved hoof explant samples (using DMEM+) and other samples that were considered autholitic and not eligible for the further experiments. Pictures are presented as Figure 3 (L 232-238 in the revised version). The other pictures have been renumbered as consequence.
- Line 203: fixed
- Line 204: fixed
- Line 205: fixed
- Line 206: fixed
- Lines 208-209: fixed
- Line 212: fixed
- Line 213: fixed
- Line 215: fixed
- Line 221: fixed
- Line 222: Authors tried to provide the suggested correction. We hope to have correctly interpreted the comments.
- Line 225: fixed
- Lines 237, 238, 241: According to Reviewer’s question: “*do the authors mean 'epidermal' instead of 'epithelial' in these instances*?” the answer is: Yes. Authors provided a correction along the text (Lines 255, 256 and 259 in the revised version).
- Lines 251-252: fixed
- Line 256: According to Reviewer’s comment: “*would avoid commenting on trends, if possible*”, only statistically significances were considered.
- Line 266-270: fixed
- Line 267: fixed

**Discussion**

- Line 274: fixed. According to Reviewer’s comment: “can the authors discuss any other papers that have attempted to characterize/use a lamellar explant model of laminitis, for the purposes of comparing their model to others? (I believe there are at least a few out there currently.)”, short sentences were added (L325-329 in the revised version).
- Line 284: fixed
- Line 290: fixed
- Line 291: fixed
- Line 301: fixed
- Line 311: fixed
- Line 315: fixed
- Line 320: fixed
- Line 321: fixed
- Line 326: fixed
- Line 333: fixed
- Line 341: fixed
- Line 342: fixed
- Line 347: fixed
- Line 348: fixed
- Line 350: fixed
- Line 351: fixed
- Line 358-359: According to Reviewer’s question “*can the authors clarify here how their approach and evaluations mitigated the effects of variable diffusion of nutrients and oxygen in explants?*” Authors added a short sentence to clarify (L380-381 in the revised version).
- Line 362: fixed
- Line 367: fixed
- Lines 367-368: According to Reviewer’s comment: “*I don't think that MMP mRNA expression necessarily correlates well with enzymatic activity, so would probably refine this statement a bit*”, the sentence has been refined.
- Lines 367-368: fixed

Reviewer 1 also requested the following modifications on Figures:

- Figure 1 - 'Sample preparation'; 'Histology/histology’: Figure 1 has been changed and designed according to the Reviewer 2 comment, considering also the corrections proposed by Reviewer 1.
- Figure 2 - 'Outer'; 'Lamellae’: Figure has been corrected according to the Reviewer’s comment
- Figure 4 - 'Histological score: Figure has been corrected according to the Reviewer’s comment
- Figures 5, 6, and 7 - 'Concentration of Methylglyoxal (mM)': all the Figures have been corrected according to Reviewer suggestion.

**Reviewer 2:**

Reviewer 2 wrote: “*Congratulations for this important research work. It is not easy to culture hoof explants of horses mainly by secondary bacteria contamination. I only suggest you to replace the figure 1 by another one more explicative and less tedious. I also suggest to add a set of photographs showing the method for explant procurement in a sequential fashion (step by step). This technique is very important and useful for equine laminitis researchers around the world*”.

Dear Reviewer, we are glad that our paper seems interesting for You. On behalf of all Authors, I would like to thank You for the time that you have spent to revise our manuscript. We accepted all Your suggestions, and here I provide the answers to Your comments.

- According to Reviewer suggestion, Figure 1 was changes. Authors hope that this new design is easier to read and more attractive for readers.
- *Pictures*: a panel of 4 pictures has been added as supplementary material in order to show the different passages of the procedure.
