# Supplementary material for: Effect of sugar metabolite methylglyoxal on equine lamellar explants: An ex vivo model of laminitis
Source: PLoS One. 2021 Jul 27;16(7):e0253840. doi: 10.1371/journal.pone.0253840 (PMC8315528; doi:10.1371/journal.pone.0253840)
Supplement: Attachment — Submitted filename: Response to Reviewers.docx [file pone.0253840.s004.docx]

**Response to Reviewers**

**Journal requirements**

Dear Editor,

As required in your email, the following items are under submission:

- Response to Reviewers
- Revised Manuscript with Track Changes
- Manuscript
- Cover letter

An updated and more precise financial disclosure statement is presented in our cover letter.

All Figures have been checked using PAGE online system and are submitted according to PAGE system revision.

Tables have been formatted according to <https://journals.plos.org/plosone/s/tables>

References have been numbered along the text in order at first citation and reference list has been updated consequently. Two references have been delated from the reference list (Bierhaus et al., 2012 and Kyaw-Tanner et al., 2012) because they were not present in-text. The in-text citation of “Visser 2008” (L199) has been removed because it does not exist. References have been organized and formatted in Vancouver style using Zotero software.

**Reviewer comments**

**Reviewer 1**

Authors would like to thank R1 for all the efforts made to improve our paper. Authors accepted all suggestions that have been addressed and corrections have been provided along the text.

Reading once again the revised manuscript, we correct some minor issue that are listed at the end of this letter.

Here we copied R1 comments (*italics*) and we provide the answers.

*Reviewer #1: The author have addressed the majority of the questions and comments that I had included in a review of the previous version of this manuscript, and I believe that it is improved. I have a few additional questions, by line, below:*

*Title - 'Effect of the sugar metabolite...'; would use* *'equine lamellar explants' instead of 'horse keratinocytes' in the title, to more accurately describe the tissue that was used; '... model of laminitis'*: corrections have been provided according to the comments.

*Abstract:*

- *Line 18 - '...and excessive levels* *absorbed into peripheral blood could be delivered to the foot and lead to...'* 🡪 correction has been provided according to Reviewer’s comment.
- *Line 23 - can remove the comma after '14*'🡪correction has been provided according to Reviewer’s comment.
- *Line 25 - can remove 'a' after 'mimicking'*🡪 correction has been provided according to reviewer’s comment.
- *Line 27-28 - this sentence (the one that begins with 'In the same...') is incomplete and should be reworded* 🡪according to reviewer’s comment the sentence has been erased. R3 suggested the same.

*Introduction:*

- *Line 41 - '...resembling the human diabetic...'*. 🡪 correction has been provided according to reviewer’s comment.
- *Line 49 - please add a space between 'from' and 'ingestion'* 🡪Correction has been provided according to reviewer’s comment.
- *Line 55 - '...caecal pH, often followed by colic and laminitis...'*. 🡪 Correction has been provided according to reviewer’s comment.
- *Line 59 - '...AGEs accumulate in significant amounts in the hoof lamellar tissue in the acute...'*. 🡪 Correction has been provided according to reviewer’s comment.
- *Line 61 - 'using the hyperinsulinemic model...'*🡪 Correction has been provided according to reviewer’s comment.
- *Line 63 - '...MG) causes the formation of AGEs, leading...'* 🡪 Correction has been provided according to reviewer’s comment.
- *Line 66 - '...intermediate, it is converted...':* 🡪 Correction has been provided according to reviewer’s comment.
- *Line 72 -* *'In equines, feeding large amounts of fermentable carbohydrates with subsequent acidosis increases the level of plasma D-lactate...'.* 🡪 Correction has been provided according to reviewer’s comment.
- *Line 75 - '...D-lactate can be considered a...'.* 🡪 Correction has been provided according to reviewer’s comment.
- *Line 84 - '...making them an attractive model...’* 🡪Correction has been provided according to reviewer’s comment.
- *Line 87 - '...of MG on equine lamellar explants in an ex vivo...'*. 🡪 Correction has been provided according to reviewer’s comment.

*Materials and Methods:*

- *Line 99 - '...typical stance, increased digital pulse, and increased hoof capsular warmth).*' 🡪 Correction has been provided according to reviewer’s comment.
- *Line 100 - '...the right fore distal limb...’* 🡪 Correction has been provided according to reviewer’s comment.
- *Line 112 - '...dermal lamellae, and the bone*. 🡪 Correction has been provided according to reviewer’s comment.
- *Line 120 - can remove 'of' in front of 'Fetal'.* 🡪 Correction has been provided according to reviewer’s comment.
- *Line 126 - 'Sample'.* 🡪 Correction has been provided according to reviewer’s comment
- *Line 142 - would use 'lamellae' instead of 'laminae' throughout the manuscript* 🡪Correction has been provided according to reviewer’s comment in this line and along the text and captions.
- *Line 143 - 'broken at'*. 🡪 Correction has been provided according to reviewer’s comment. Also, R3 suggested the same correction.
- *Line 149 - 'One sample from each time point and from each MG concentration...'*. 🡪 Correction has been provided according to reviewer’s comment.
- *Line 150 - would add a comma after 'paraffin'; '...slices were mounted...'.* 🡪 Correction has been provided according to reviewer’s comment.
- *Line 153 - '...microscope, and images were captured...'.* 🡪 Correction has been provided according to reviewer’s comment.
- *Line 160 - '...evaluated for lesions of the secondary...'*. 🡪 Correction has been provided according to reviewer’s comment.
- *Line 164 - 'RNA-Later solution (Ambion), and the stratum lamellatum was disrupted using...'* 🡪 Correction has been provided according to reviewer’s comment. R3 suggested ‘RNAlater’ instead of 'RNA-Later’. We checked the manufactured label, and we wrote ‘RNAlater’.
- *Line 177 - '...MMP-14, and TIMP-2 transcripts, qPCR...'.* 🡪 Correction has been provided according to reviewer’s comment.
- *Line 179 - 'designed from Equus caballus...'.* 🡪Correction has been provided according to reviewer’s comment.
- *Line 200 - '...were designed from Equus caballus...'*. 🡪 Correction has been provided according to reviewer’s comment.
- *Line 205 - 'one-way ANOVA and Tukey's multiple....* 🡪. Correction has been provided according to reviewer’s comment.
- *Line 207 - '...their distributions were analyzed using two-way ANOVA...*'. 🡪 Correction has been provided according to reviewer’s comment.

*Results and discussion:*

- *Line 221 - '...all structures; all samples were...*'. 🡪 Correction has been provided according to reviewer’s comment.
- *Line 225 -* *'characteristics'.* 🡪 Correction has been provided according to reviewer’s comment.
- *Line 236 - 'predetermined'*. 🡪 Correction has been provided according to reviewer’s comment.
- *Line 245 - '...for 48 h can be observed*. 🡪 Correction has been provided according to reviewer’s comment.
- *Line 258 - '...of all layers and were statistically...*' 🡪Correction has been provided according to reviewer’s comment.
- *Line 267 - 'concentrations'*. 🡪 Correction has been provided according to reviewer’s comment.
- *Line 280 - can remove the comma after 'concentration'*. 🡪 Correction has been provided according to reviewer’s comment.
- *Line 296 - '...as a suitable viable model system for investigating...*’ 🡪 Correction has been provided according to reviewer’s comment.
- *Line 307 - 'with our results, FBS and...*' 🡪Correction has been provided according to reviewer’s comment.
- *Line 309 - '...period, supporting the viability of the samples...*' 🡪 Correction has been provided according to reviewer’s comment.
- *Line 310 - 'Other papers have evaluated the possibility of using hoof explant samples but focus their attention on different metabolic goals (e.g.,...'*. 🡪 Correction has been provided according to reviewer’s comment.
- *Line 312 - 'To the authors' knowledge, this is the first paper focusing specifically on the role of MG (...'* 🡪 Correction has been provided according to reviewer’s comment.
- *Line 315 - 'to modification of the hoof lamellae...*’ 🡪 Correction has been provided according to reviewer’s comment.
- *Line 316 - '...that untreated samples are able to bear...'*. 🡪 Correction has been provided according to reviewer’s comment.
- *Line 317 - '...confirmed that higher concentrations of MG and a longer...*' 🡪 Correction has been provided according to reviewer’s comment.
- *Line 319 - '...were confirmed by histological...'.* 🡪. Correction has been provided according to reviewer’s comment.
- *Line 322 - 'The results of gene expression of MMPs*...' 🡪 Correction has been provided according to reviewer’s comment.
- *Line 325 - '...matrix remodeling, more severe histological features, and...*' 🡪 Correction has been provided according to reviewer’s comment.
- *Line 337 - '...starch overload or other causes, increases...*' 🡪 Correction has been provided according to reviewer’s comment.
- *Line 346 - can remove the comma after 'MMP2' here.* Correction has been provided according to reviewer’s comment.
- *Line 349 - 'yielding*' 🡪Correction has been provided according to reviewer’s comment.
- *Line 363 - '...various pathologies; for example,...*' 🡪 Correction has been provided according to reviewer’s comment
- *Line 370 - '...not sufficient to induce laminitis in vitro.*' 🡪. Correction has been provided according to reviewer’s comment.
- *Line 373-374 - '...more likely contributing to the disease's...*' 🡪 Correction has been provided according to reviewer’s comment.
- *Line 383-385 - '...(Breton horses) that were relatively young; it would be interesting to apply the same ex vivo model of laminitis to even older horses of different breeds, such as pony breeds, to assess any differences...'.* 🡪 Corrections have been provided according to reviewer’s comment.
- *Line 388 - '...could be used for ex vivo studies*.' 🡪 Correction has been provided according to reviewer’s comment.
- *Line 393 - would use 'supported' instead of 'confirmed' here*. 🡪 Correction has been provided according to reviewer’s comment.

Supplemental data:

- *Line 519 - would use 'lamellar' instead of 'laminar' here*. 🡪The correction has been provided according to the comment in L519 and in Fig S1.

**Reviewer 2**

*Reviewer #2: Congratulations. This is an important research work. In general, you addressed the concerns raised during the review process.*

Author would like to sincerely thank R2 for all the efforts made to improve our paper. We are pleased to hear that R2 is satisfied about the corrections that have been provided in the first revision round.

Reading once again the revised manuscript, we correct some minor issue that are listed at the end of this letter.

**Reviewer 3**

Authors would like to thank R3 for all the efforts made to improve our paper. Authors accepted all suggestions that have been addressed and corrections have been provided along the text.

Reading once again the revised manuscript, we correct some minor issue that are listed at the end of this letter.

Here we copied R3 comments (*italics*) and we provide the answers.

*Reviewer #3: In this paper, the authors are examining the effects of excessive methylglyoxal on the hoof lamellar structure with an aim to identify the mechanism behind the Laminitis disease. They use an ex vivo experimental design and perform various types of analysis - macroscopic analysis, histological analysis, separation force test and gene expression. They identify that high levels of methylglyoxal could induce irreversible damage in the hooves which mimicks laminitis in an ex vivo model.*

*Overall, the findings of the manuscript are well-supported by the data and the methods used are appropriate. The authors have also addressed the reviewers comments adequately. However, there are some points to consider that I have outlined below:*

1. *In the revised manuscript, the authors have renamed the ‘Results’ section as ‘Results and Discussion’, however, the discussion has not been incorporated with the results. It is still presented as a separate section (minus the heading). The layout of this section will have to be changed significantly if the authors want to keep the ‘Results and Discussion’ heading. If not, I would recommend adding the ‘Discussion’ heading and keeping ‘Results’ separate. 🡪* Thank you for your suggestions. Authors would like to maintain the two sections separated. For this reason, headings have been changed.

*Minor concerns:*

*1. In the abstract, the authors mention ‘Microscopical and histological analysis’, however, in the rest of the manuscript, there is ‘Macroscopical analysis’. Please correct accordingly* 🡪 According to reviewer’s comment, correction has been provided.

*2. In the abstract, the second last line starting with ‘In the same samples…’ (Line 29), does not make sense, it seems incomplete. Please edit.* 🡪 the sentence has been edited.

*3. Line 45, ‘…influences and weight/ pressures…’ needs to be edited for coherence*. 🡪 The sentence has been edited according to reviewer’s comment.

*4. Lines 48-51, need to be changed to make sense. Right now, it seems like a collection of terms*. 🡪 sentence has been rephrased in order to be clearer

*5. Line 57, ‘this means’ should be ‘which means’.* 🡪 Correction has been provided according to reviewer’s comment.

*6. Line 64, ‘hoof lamellar tissue level’, the word ‘level’ can be removed.* 🡪 ‘Level ‘has been removed, also according to R1 suggestion.

7. *Line 70, ‘highly reactive intermediate is’, should be ‘highly reactive intermediate, it is’.* 🡪 The same correction was proposed also by R1. Correction has been provided.

*8. Line 78, ‘sequent acidosis’, do the authors mean ‘subsequent acidosis’?* 🡪 yes, thank you for your comment, Correction has been provided.

*9. Line 79, ‘increase the level of plasma levels’, should be ‘increase the plasma levels of’*.🡪Correction has been provided according to reviewer’s comment.

*10. Line 81, ‘also because’ is redundant. Use either also, or because*. 🡪 Correction has been provided according to reviewer’s comment.

*11. Line 89, ‘attracting model’ should be ‘attractive model*’. 🡪The same correction was proposed also by R1. Correction has been provided.

*12. Line 100, ‘for this study purpose’ can be rephrased to, ‘for the purposes of this study’*. 🡪 The sentence has been rephrased according to the comment of R3.

*13. Line 105, ‘typical pain position and digital pulse and hot hoof’, one ‘and’ can be removed*. 🡪 R1 asked to rephrase the sentence. We hope that R3 agrees with this version.

*14. Line 107, ‘within 45 minutes of slaughter’ should be ‘within 45 minutes’, since the sentence is starting with ‘following slaughter’*. 🡪 thank you for your comment, Correction has been provided.

*15. Line 114, Mungall and Pollitt is missing an in-text citation*. 🡪 All the in-text citations have been formatted according to the journal requirements and the reference list has been updated. According to this, also the comment about L114 has been fixed.

*16. Line 131, should be ‘Fig S1’ to make it consistent with the rest of the manuscript.* 🡪 correction has been provided. Thank you for the suggestion.

*17. Line 133, do the authors mean ‘Sample survival’?* 🡪 The same correction was proposed also by R1. Correction has been provided.

*18. Line 141, for ‘controls (K)’, reference the figure number*. 🡪 reference to figures has been added

*19. Line 144, ‘the’ before structural integrity should be removed.* 🡪 ‘the’ has been removed

*20. Line 150, do the authors mean ‘broken’, instead of ‘brocken’*?--> yes, thank you for your suggestion.

*21. Line 172, change RNA later to RNAlater (as per manufacturers label).* 🡪 R1 proposed a different correction (‘RNA-Later’) but we checked the manufacturer label and we accepted your suggestion.

*22. Line 198 and Line 202, move Cq definition from line 202 to line 198*. 🡪 The Cq definition has been moved and the sentences have been rephrased in order to be clearer.

*23. Line 207, table 1 is not in line with the text*. 🡪 all tables have been formatted according to the journal style. We hope that table 1 now is in line with the text.

*24. Line 211-214, do not make sense. Which softwares? Which companies? Please edit. Line 211, remove ‘a’ before ‘commercially’ if you are referring to more than 1 software.* 🡪 thank you for your suggestion. The sentence has been edited and corrections have been provided.

*25. Line 216, ‘Turkey’s’ should be ‘Tukey’s’*. 🡪 Correction has been provided.

*26. Line 222, define SD*. 🡪 SD has been defined.

*27. Line 228, should be ‘performed for all samples’*. 🡪 thank you for your suggestion. Correction has been provided.

*28. Line 232-233, separate the sentences. ‘All samples’ should be a different sentence*. 🡪 R1 asked to change ‘,’ with ‘;’ to separate the sentences. We hope that R3 agrees with R1.

*29. Line 248, should be ‘predetermined’*. 🡪 The same correction was proposed also by R1. Correction has been provided.

*30. Line 254-255 is very confusing. Please clarify*. 🡪 these two lines have been modified according to the comments provided by reviewers in the fist round. Nevertheless, Authors checked the first manuscript that was submitted and tried to rephrase the sentences in order to be clearer and more related to the original meaning.

*31. Line 271-272, ‘and resulted statistically different’, please clarify*. 🡪 sentence has been rephrased according to R1 comment. We hope that R3 agrees with R1.

*32. Line 277, what do you mean by definition?* 🡪 no, in this case, we mean the definition of the histological image.

*33. Line 280, should be ‘concentrations’*. 🡪 ‘s’ has been added according to R1 and R3comments.

*34. Fig 6-8 legend, the gene descriptions should be in the text and not in the figure legends.* 🡪 the definitions have been removed from figure captions.

*35. Line 328-329, please rephrase for coherence.* 🡪 some corrections have been provided according to the R1 comments. We hope that R3 agrees with R1.

*36. Line 330, should be ‘In our knowledge’*. 🡪🡪some corrections have been provided according to the R1 comments. We hope that R3 agrees with R1.

*37. Line 340, please edit for coherence*. 🡪 some corrections have been provided according to the R1 comments. We hope that R3 agrees with R1.

*38. Line 344, edit spelling to ‘correlated’.* 🡪 thank you. Correction has been provided.

*39. Line 345, should be ‘resulting in structural weakness’*. 🡪thank you for your comment. Correction has been provided.

*40. Line 367, please define the ‘entire process’ so as to make the sentence complete.* 🡪 we add ‘laminitic’

*41. Line 369, edit spelling to ‘yielding’.* 🡪 also R1 suggested this correction. Correction has been provided.

*42. Line 378, the acronym AGEs does not need to be defined again.* 🡪 definition has been removed.

*43. Line 391, the word ‘bacteria’ should be before E.coli and not after*.--> Thank you for your comment. This correction has been fixed.

*44. Line 395, rephrase for coherence*. 🡪 also R1 suggested a rephrasing here. We hope that R3 agrees with R1.

*45. Line 403-406, rephrase for coherence*. 🡪 also R1 suggested a rephrasing here. We hope that R3 agrees with R1.

*46. Line 410, remove ‘an’ before ex vivo.* 🡪 ‘an’ as been delated according to R1 and R3 suggestions.

*47. Line 412, time-dependent would be more appropriate than ‘time-related’.* 🡪 the word has been changed according to R3 suggestion.

*48. Figure 1, The authors can consider changing ‘force test’ to ‘separation force test’. It would then be consistent throughout the manuscript.* 🡪 Figure 1 has been changed according to R3 suggestion.

*49. Figure 4, table headings are missing.* 🡪 the complete table has been inserted.

*50. Figure 4,6,7,8, the legend for time needs to be consistent throughout. Either 24h, 48h or T24h, T48h*. 🡪 Fig. 4,6-8 are now consistent and the same wording (T24 h and T 48 h) has been used along the text.

*51. Figure 6-8, the Y-axis should be Cq instead of Ct, for consistency between figures and text*. 🡪 Y axis label has been corrected according to R3 comment.

**Authors comments**

- Reading once again the manuscript, we have found some typos and double-spaced words: we have fixed everything.
- Some references have been delated. An explanation is provided at the beginning of this letter and in the cover letter.
- L292: Fig 9 does not exist. Thus a correction has been provided.
- L305: ‘/maintaing’ has been removed.
- L356: ‘ alpha oxoaldehydes’ instead of ‘alphaoxoaldehydes’
- In the whole document, MMP-2, MMP-14 and TIMP-2 (using ‘-‘) instead of MMP2, MMP14 and TIMP2 have been written. This was done in order to be coherent along the text, tables and figures using the same wording and according to a suggestion of the first round of revision.
- Table 2: the title was missing. We added it.
